# Supplementary material for: Specificity, synergy, and mechanisms of splice-modifying drugs
Source: Nat Commun. 2024 Feb 29;15:1880. doi: 10.1038/s41467-024-46090-5 (PMC10904865; doi:10.1038/s41467-024-46090-5)
Supplement: Supplementary file 3 — Inventory of Supporting Information [file 41467_2024_46090_MOESM3_ESM.pdf]

Inventory of Supporting Information

Manuscript #: NCOMMS-23-08099A
Corresponding author name(s): Adrian R. Krainer, Justin B. Kinney

1. Supplementary Information

A. PDF Files

Item	Present?	Filename<br>Whole original file name including extension. i.e.: Smith_SI.pdf. The extension must be .pdf	A brief, numerical description of file contents.<br>i.e.: *Supplementary Figures 1-4, Supplementary Discussion, and Supplementary Tables 1-4.*
Supplementary Information	Yes	22_drugs_v18_si.pdf	Experimental methods, Data processing, exploratory analysis, and molecular dynamics simulations, Mathematical model definitions, Bayesian model inference, Supplemental Tables S1-S4, Supplemental Figures S1-S14, Supplemental References
Reporting Summary	Yes	v18_reporting_summary.pdf	
Peer Review Information	No	*OFFICE USE ONLY*	

3. Source Data

Parent Figure or Table	Filename<br>Whole original file name including extension. i.e.: *Smith_SourceData_Fig1.xls,* or *Smith_ Unmodified_Gels_Fig1.pdf*	Data description<br>i.e.: Unprocessed western Blots and/or gels, Statistical Source Data, etc.
Supplementary Information Fig. S1I	supporting_data_fig_S1I_uncropped.jpg	Uncropped radioactive RT-PCR gel image
