# Supplementary material for: Specificity, synergy, and mechanisms of splice-modifying drugs
Source: Nat Commun. 2024 Feb 29;15:1880. doi: 10.1038/s41467-024-46090-5 (PMC10904865; doi:10.1038/s41467-024-46090-5)
Supplement: Supplementary file 4 — Reporting Summary [file 41467_2024_46090_MOESM4_ESM.pdf]

Corresponding author(s): Adrian R. Krainer, Justin B. Kinney

Last updated by author(s): YYYY-MM-DD

Reporting Summary

Nature Portfolio wishes to improve the reproducibility of the work that we publish. This form provides structure for consistency and transparency in reporting. For further information on Nature Portfolio policies, see our Editorial Policies and the Editorial Policy Checklist.

Statistics

For all statistical analyses, confirm that the following items are present in the figure legend, table legend, main text, or Methods section.

n/a	Confirmed	
☐	☒	The exact sample size (*n*) for each experimental group/condition, given as a discrete number and unit of measurement
☐	☒	A statement on whether measurements were taken from distinct samples or whether the same sample was measured repeatedly
☐	☒	The statistical test(s) used AND whether they are one- or two-sided<br>*Only common tests should be described solely by name; describe more complex techniques in the Methods section.*
☒	☐	A description of all covariates tested
☒	☐	A description of any assumptions or corrections, such as tests of normality and adjustment for multiple comparisons
☐	☒	A full description of the statistical parameters including central tendency (e.g. means) or other basic estimates (e.g. regression coefficient) AND variation (e.g. standard deviation) or associated estimates of uncertainty (e.g. confidence intervals)
☒	☐	For null hypothesis testing, the test statistic (e.g. *F*, *t*, *r*) with confidence intervals, effect sizes, degrees of freedom and *P* value noted<br>*Give P values as exact values whenever suitable.*
☐	☒	For Bayesian analysis, information on the choice of priors and Markov chain Monte Carlo settings
☒	☐	For hierarchical and complex designs, identification of the appropriate level for tests and full reporting of outcomes
☐	☒	Estimates of effect sizes (e.g. Cohen's *d*, Pearson's *r*), indicating how they were calculated

Our web collection on statistics for biologists contains articles on many of the points above.

Software and code

Policy information about availability of computer code

Data collection	RNA-seq raw data was obtained by NextSeq 2000 (150-bp, paired-end).<br>RT-qPCR data was obtained by QuantStudio 6 Flex system.
Data analysis	Quality control on RNA-seq was done using FastQC v0.11.9, RSeQC v4.0.0 and multiqc 1.0.dev0.<br>Reads were mapped against the hg38 and Ensembl 103 annotation using STAR v2.7.8a. Annotation transformations were done with gffread v0.12.1.<br>Alternative splicing analysis was done using rMATS 4.1.0 and custom scripts for statistical analysis.<br>MPSA analysis and bayesian model fitting was done with custom scripts.<br>Custom scripts and code for reproducing results and figures are provided here: https://github.com/jbkinney/22_drugs

For manuscripts utilizing custom algorithms or software that are central to the research but not yet described in published literature, software must be made available to editors and reviewers. We strongly encourage code deposition in a community repository (e.g. GitHub). See the Nature Portfolio guidelines for submitting code & software for further information.

Data

Policy information about availability of data

All manuscripts must include a data availability statement. This statement should provide the following information, where applicable:

- Accession codes, unique identifiers, or web links for publicly available datasets
- A description of any restrictions on data availability
- For clinical datasets or third party data, please ensure that the statement adheres to our policy

MPSA and RNA-seq data have been deposited in the NCBI GEO database (accession number GSE221868).

Research involving human participants, their data, or biological material

Policy information about studies with human participants or human data. See also policy information about sex, gender (identity/presentation), and sexual orientation and race, ethnicity and racism.

Reporting on sex and gender	Not applicable
Reporting on race, ethnicity, or other socially relevant groupings	Not applicable
Population characteristics	Not applicable
Recruitment	Not applicable
Ethics oversight	Not applicable

Note that full information on the approval of the study protocol must also be provided in the manuscript.

Field-specific reporting

Please select the one below that is the best fit for your research. If you are not sure, read the appropriate sections before making your selection.

☒ Life sciences ☐ Behavioural & social sciences ☐ Ecological, evolutionary & environmental sciences

For a reference copy of the document with all sections, see nature.com/documents/nr-reporting-summary-flat.pdf

Life sciences study design

All studies must disclose on these points even when the disclosure is negative.

Sample size	No statistical methods were used to predetermine sample size. Sample sizes were chosen based on experimental resources.
Data exclusions	No data were excluded.
Replication	Replicate experiments, performed as described in the text, were used to replicate experiments. All replication attempts were successful.
Randomization	Our study did not involve any randomization because our study did not involve the allocation of individuals to experimental groups.
Blinding	Our study did not involve any blinding because our study did not involve the allocation of individuals to experimental groups.

Reporting for specific materials, systems and methods

We require information from authors about some types of materials, experimental systems and methods used in many studies. Here, indicate whether each material, system or method listed is relevant to your study. If you are not sure if a list item applies to your research, read the appropriate section before selecting a response.

Materials & experimental systems

n/a	Involved in the study
☒	☐ Antibodies
☐	☒ Eukaryotic cell lines
☒	☐ Palaeontology and archaeology
☒	☐ Animals and other organisms
☒	☐ Clinical data
☒	☐ Dual use research of concern
☒	☐ Plants

Methods

n/a	Involved in the study
☒	☐ ChIP-seq
☒	☐ Flow cytometry
☒	☐ MRI-based neuroimaging

Eukaryotic cell lines

Policy information about cell lines and Sex and Gender in Research

Cell line source(s)	HeLa cells: ATCC
Authentication	HeLa cells were authenticated by STR profiling at an external facility (Genetics core, University of Arizona)
Mycoplasma contamination	Cells were tested each month for mycoplasma testing and have tested negative.
Commonly misidentified lines (See ICLAC register)	None

Plants

Seed stocks	No plants were used in our study.
Novel plant genotypes	No plants were used in our study.
Authentication	No plants were used in our study.
